# Supplementary material for: Transactivation of miR-202-5p by Steroidogenic Factor 1 (SF1) Induces Apoptosis in Goat Granulosa Cells by Targeting TGFβR2
Source: Cells. 2020 Feb 14;9(2):445. doi: 10.3390/cells9020445 (PMC7072820; doi:10.3390/cells9020445)
Supplement: Supplementary file 1 [file cells-09-00445-s001.pdf]

Supplementary Files

Article

Transactivation of miR-202-5p by Steroidogenic Factor 1 (SF1) Induces Apoptosis in Goat Granulosa Cells by Targeting TGFβR2

Qiang Ding †, Miaohan Jin †, Yaoyue Wang, Jiao Liu, Peter Kalds, Ying Wang, Yuxin Yang, Xiaolong Wang * and Yulin Chen *

Key Laboratory of Animal Genetics, Breeding and Reproduction of Shaanxi Province, College of Animal Science and Technology, Northwest A&F University, Yangling 712100, Shaanxi, China; dingqiang@nwafu.edu.cn (Q.D.); jinmiaohan@nwafu.edu.cn (M.J.); 18792629437@163.com (Y.W.); liujiaodk@163.com (J.L.); peterkalds@nwafu.edu.cn (P.K.); nd2013wang@163.com (Y.W.); yangyuxin2002@126.com (Y.Y.)

† These authors equally contribute to this paper

* Correspondence: xiaolongwang@nwafu.edu.cn (X.W.); chenyulin@nwafu.edu.cn (Y.C.)

Supplementary Tables

Table S1. the primers are used in this study.

Primers	Sequences（5′–3′）
CYP19A1 promoter R	TCCTCGAGTGGAGATCAGAAGAGTGGAA
No SF1 binding site (–40 nt to –211nt)	CTGCTAGCTCTTGGGCTTGCTTGTTT
0.5 kb promoter CYP19A1 (–40 nt to –522 nt)	TAGCTAGCTGACTGGAGAAGCCTACC
1.6 kb promoter CYP19A1 (–40 nt to –1640 nt)	CGGCTAGCGTGGTGACAGATAACAACTAG
CYP19A1 Promoter SF1 binding site mut F	ACTCTACACCGTACCGAAATGCTGCACTTCAAGCCAAAAG
CYP19A1 promoter SF1 binding site mut R	ATTTCGGTACGGTGTAGAGTCTCAGGTTCGTTTAGACGCTTG
miR-202 WT promoter R	CCAAGCTTCTTACCTGGTCGCAGTCA
miR-202 WT 0.5 K promoter F (–130 nt to –515 nt)	GAAGATCTGAGATGCCGTTCCTGAAG
miR-202 NO binding site promoter F (–130nt to –257nt)	GAAGATCTGCCTGGCATAAAAAGGCGCCA
miR-202 promoter SF1 binding site mut F	CCGCGATTGAATCTGCCTGGCATAAAAAGGCGCCAGGCGGTC
miR-202 promoter SF1 binding site mut R	AGGCAGATTCAATCGCGGGGCGGCCGTCACGTGCGGTCAC
TGFβR2 F	TCAGCCGAATAGGTAGGAA
TGFβR2 R	AGAGGTCAATGAGCAACAG
TGFβR1 F	TCCACGAGACAGGCCATTTG
TGFβR1 R	GACGGCTTTCCTGTAAAAGGG
GAPDH F	ACACCCTCAAGATTGTCAGC
GAPDH R	ATAAGTCCCTCCACGATGC
BAX F	CTGAGCGAGTGTCTGAAG
BAX R	TCGAAGGAAGTCCAATGTC
Bcl2 F	TTCTTTGAGTTCGGAGGGG
Bcl2 R	CCAGGAGAAATCAAACAGGGGC
PCNA F	ACTCGTCTCATGTCTCCTT
PCNA R	GGTGTCCGCATTATCTTCA
Notch2 F	TGGAGATGAAGAACAGGAAG
Notch2 R	AACAGCAAGCAGATAGAGAA
Chi-miR-202-5p for cDNA reverse	GTCGTATCCAGTGCAGGGTCCGAGGTATTCGCACTGGATACGACA AAGAA
Chi-miR-202-5p for qPCR	GCGGGCTTCCTATGCATATACT
UR R	CAGTGCAGGGTCCGAGGT
Cel-miRNA-39 R	GTCGTATCCAGTGCAGGGTCCGAGGTATTCGCACTGGATACGACC AAGCT
Cel-miRNA-39 F	TCACCGGGTGTAAATCAGCT
U6 F	CTCGCTTCGGCAGCACA
U6 R	AACGCTTCACGAATTTGCGT
TGFβR2 3′UTR WT F	CCCTCGAGACTCCAAGAAGTTGTTGTCA
TGFβR2 3′UTR WT R	TTTGCGGCCGCTCCACCTGAACCGAGAAG
TGFβR2 3′UTR Wut F	TAATCCTCGAGATGGAACTTGGTAATCCACAAACA
TGFβR2 3′UTR Wut R	TCCATCTCGAGGATTAAAGCTCATTCTTGGTT
SF1 CDS F	CCAAGCTTATGGACTATTCGTACGACGA
SF1 CDS R	CGGGATCCAGTCTGCTTGGCCTGCAGCA
TGFβR2 CDS F	CCAAGCTTATGGGCCGGGGGCTGCTC
TGFβR2 CDS R	CGGGATCCCTATTTGGTAGTGTTGAGGG
ChIP CYP19A1 F	GAGAACTCAGTCACTCTACC

ChIP CYP19A1 R	TGGACACTTAGACAAGACAA
ChIP miR-202 F	CTCAGAGCAGGTCTTTGTG
ChIP miR-202 R	CTTACCTGGTCGCAGTCA

Table S2. the sequences of siRNA are used in this study.

siRNA	sense（5′–3′）	antisense（5′–3′）
TGFBR2-goat-984	GCAAUGACCACAUCAUCUUTT	AAGAUGAUGUGGUCAUUGCTT
TGFBR2-goat-1843	CCUCGAGUCCAGGAUGAAUTT	AUUCAUCCUGGACUCGAGGTT
NR5A1-goat-311	GCACGGUGCAGAACAACAATT	UUGUUGUUCUGCACCGUGCTT
NR5A1-goat-525	GGCACAGAUUCGAGCCAAUTT	AUUGGCUCGAAUCUGUGCCTT
chi-miR-202-5p mimics	UUCCUAUGCAUAUACUUCUUU	AGAAGUSUGCAUAGGAAUU
chi-miR-202-5p inhibitor	AAAGAAGUAUGCAUAGGAA	
mimics NC/siRNA NC	UUCUCCGAACGUGUCACGUTT	ACGUGACACGUUCGGAGAATT
Inhibitor NC	CAGUACUUUUGUGUAGUACAA	

Supplementary Figure

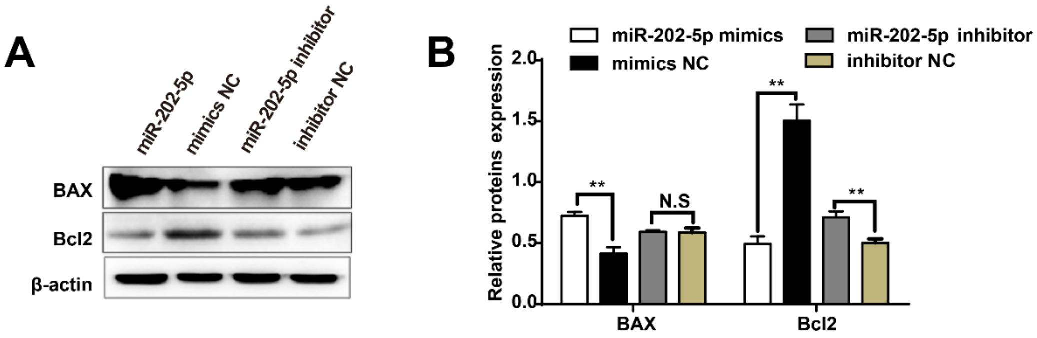

Figure S1. miR-202-5p induces GCs apoptosis. GCs were treated with miR-202-5p mimics or miR-202-5p inhibitors and mimics NC or inhibitor NC. Proteins levels of BAX and Bcl2 detected by WB (**A**); The relative protein levels were calculated (**B**).
